# Host‐ and pathogen‐derived adjuvant coatings on protein nanoparticle vaccines

**DOI:** 10.1002/btm2.10052

**Published:** 2017-02-03

**Authors:** Timothy Z. Chang, Ishatou Diambou, Jong Rok Kim, Baozhong Wang, Julie A. Champion

**Affiliations:** ^1^ School of Chemical and Biomolecular Engineering Georgia Institute of Technology Atlanta GA 30332; ^2^ Institute for Biomedical Sciences Georgia State University Atlanta GA 30332

**Keywords:** adjuvant, affinity maturation, coating, flagellin, immunoglobulin M, nanoparticle, vaccine

## Abstract

Nanoparticulate and molecular adjuvants have shown great efficacy in enhancing immune responses, and the immunogenic vaccines of the future will most likely contain both. To investigate the immunostimulatory effects of molecular adjuvants on nanoparticle vaccines, we have designed ovalbumin (OVA) protein nanoparticles coated with two different adjuvants—flagellin (FliC) and immunoglobulin M (IgM). These proteins, derived from *Salmonella* and mice, respectively, are representatives of pathogen‐ and host‐derived molecules that can enhance immune responses. FliC‐coated OVA nanoparticles, soluble FliC (sFliC) admixed with OVA nanoparticles, IgM‐coated nanoparticles, and OVA‐coated nanoparticles were assessed for immunogenicity in an *in vivo* mouse immunization study. IgM coatings on nanoparticles significantly enhanced both antibody and T cell responses, and promoted IgG2a class switching but not affinity maturation. FliC‐coated nanoparticles and FliC‐admixed with nanoparticles both triggered IgG2a class switching, but only FliC‐coated nanoparticles enhanced antibody affinity maturation. Our findings that affinity maturation and class switching can be directed independently of one another suggest that adjuvant coatings on nanoparticles can be tailored to generate specific vaccine effector responses against different classes of pathogens.

## 1 Introduction

Nanoparticle vaccine delivery systems have emerged as an attractive means of enhancing subunit vaccine adjuvancy. Particulate vaccine carriers can control release of soluble antigens to the immune system and protect them from degradation.^1^ However, nanoparticles have been found to be more than just passive antigen depots, and certain types of particles exhibit their own immunostimulatory effects on antigen presenting cells. The exact nature of this nanoparticulate‐mediated adjuvancy is unknown, and many fundamental studies have examined the immunological effects of nanoparticle properties such as size,^2^ surface charge,^3^ shape,^4^ and material.^5^ Generalized vaccine particle design principles are difficult to elucidate from these studies, however, due to our incomplete understanding of immunology of vaccination, and specifically the type of immune response needed to successfully vaccinate against a particular pathogen.^6^

The molecular adjuvants are a more predictable class of immunostimulants. Pathogen‐associated molecular patterns (PAMPs) are macromolecules that interact with specific pattern recognition receptors (PRRs) on or inside antigen presenting cells.^1^, ^7^ Receptors that bind bacterially‐derived or virally‐derived macromolecules are hypothesized to initiate adaptive immune responses geared toward those particular classes of pathogens.^7^, ^8^ Toll‐like receptors (TLRs) are a class of membrane‐bound PRRs that have been extensively studied for vaccine adjuvant use.^9^, ^10^, ^11^ However, safety concerns over administration of pathogen‐derived compounds require thorough investigation.^12^ Currently, several pathogen‐derived vaccine adjuvants are undergoing clinical trials, but only two have been approved for use in humans.^13^

Flagellin (FliC) is a TLR‐5 ligand shown to greatly enhance responses to influenza vaccination.^14^, ^15^ Given the strength of FliC as an adjuvant, vaccines have been proposed with genetic fusion of antigenic peptides with the FliC protein,^11^, ^16^ as well as nanoparticles decorated with FliC.^17^, ^18^ As of this writing, at least six clinical trials have been completed with FliC‐fusion proteins.^19^ The propensity of certain FliC‐fusion proteins to aggregate, even at 4°C, may decrease their efficacy,^11^ and the sequence‐dependent nature of FliC‐fusion protein stability reduces its attractiveness as a platform technology. Nanoparticles with a stable, native FliC coat, or with native FliC admixed can combine the immunostimulatory properties of FliC with those of antigen‐containing nanoparticles. The optimal location of antigen and adjuvant in nanoparticle vaccine formulations is still under active research,^9^, ^20^ and recent findings suggest that flagellated bacteria in the gut assist in TLR‐5‐mediated adjuvancy to subcutaneously administered influenza vaccines.^14^ Using TLR ligands as adjuvants, however, poses the risk of safety issues^11^ and immune responses against the adjuvant itself.^21^

The use of host‐derived proteins as vaccine adjuvants may be able to address some of the issues associated with pathogen‐derived adjuvants. Antibodies, or immunoglobulins (Ig), coat pathogens during the immune response to an infection, and these proteins may be able to act as *in situ* adjuvants rendering nanoparticles more immunogenic *in vivo*. While antibodies immobilized by affinity interactions on the nanoparticles' surface should remain bound, any soluble Ig in the formulation should be recognized as host protein and consequently nonimmunogenic, and would simply enter the host's circulating repertoire of antibodies. Additionally, the current, widespread good manufacturing practice production of humanized antibodies offers a pathway for large‐scale production of immunoglobulin‐based adjuvants.

The idea of immunoglobulin‐mediated adjuvancy has been explored through the use of antibody‐bound antigen, or immune complexes, as vaccines.^22^, ^23^, ^24^, ^25^ IgG2a complexed with soluble ovalbumin (OVA) was able to enhance specific anti‐OVA antibody concentrations and CD4^+^ T cell responses by over an order of magnitude in comparison to soluble OVA.^26^ Although several sources state that immunoglobulins enhance responses against soluble antigen and suppress them when bound to particulates,^27^ this assertion was based on evidence of anti‐Rh factor IgG suppressing immune responses against fetal erythrocytes in pregnant women.^28^ Immunosuppressive responses against IgG‐opsonized nanoparticulates have not been definitively reported. Moreover, a study comparing the inflammatory properties of soluble and insoluble immune complexes from rheumatoid synovial fluid found that the larger, insoluble immune complexes were more immunostimulatory than the soluble ones,^24^ supporting the hypothesis that particle size and immunoglobulin opsonization may synergistically enhance immune responses.

The protein corona that forms on nanoparticles in serum in vivo consists of many protein types, and biomaterial‐serum protein interactions are an active area of research.^29^ Engineering biomaterial surfaces to bind antibodies can enhance immunogenicity by targeting the antigen particles to macrophages and dendritic cells via Fc receptors on these antigen‐presenting cell types.^30^ Furthermore, antibody‐opsonized nanoparticles and microparticles provide a unique platform for activating the complement system, an inflammatory extracellular signaling cascade designed to neutralize infection, trigger local inflammation, and assist in the adaptive immune response.^7^, ^31^

The present study of adjuvant nanoparticle coatings looks at both pathogen‐derived flagellin (FliC) and the host‐derived antibody immunoglobulin M (IgM). IgM is the first antibody isotype made by antibody‐producing B cells and is a stronger activator of the complement system than the more prevalent IgG.^32^ It is possible that IgM enhances the adaptive immune response to the antigen to which it is bound. Given its lower affinity and different Fc structure than the more prevalent IgG, IgM likely serves an immunoregulatory function in addition to any neutralizing capabilities it may have. Although it has been proposed as a potential vaccine adjuvant due to its interactions with complement, B cells and T cells,^33^ to the best of our knowledge, IgM has not been tested as part of any vaccine formulation yet.

Our vaccine nanoparticle core consists of model OVA protein nanoparticles (PNPs), which are nanoparticles composed entirely of cross‐linked antigen protein.^10^, ^34^ Our immunization of mice with FliC‐ and IgM‐coated OVA PNPs examines (a) whether IgM could be used as a host‐derived vaccine adjuvant, and (b) whether pathogen‐derived adjuvants were more effective bound or unbound from antigen nanoparticles. Overall, our immunization study profiled differences in host‐ and pathogen‐derived adjuvant responses.

## 2 Methods

### 2.1 Materials

Endotoxin‐free EndoFit™ OVA was dissolved in sterile phosphate‐buffered saline (PBS) for all nanoparticle formulations administered in vivo. OVA and endotoxin‐free OVA were purchased from Invivogen (San Diego, CA). Antibodies were purchased from Thermo Fisher Scientific (Rockford, IL) unless stated otherwise.

### 2.2 FliC expression and purification

The plasmid pET22b‐flic was used to express recombinant FliC from *Salmonella typhimurium*.^35^ The plasmid was transformed into *E. coli BL21* for expression. Transformed *E. coli* were grown in 1‐L Luria Bertani broth with 100 μg/ml ampicillin from 10 ml overnight cultures. Expression was induced after approximately 2 hr (OD_600_ ≈ 0.6) with 0.25 mM isopropyl β‐d−1‐thiogalactopyranoside (IPTG). Recombinant FliC was expressed over 24 hr and purified using native Ni‐affinity purification according to the manufacturer's instructions (Ni‐NTA agarose, Qiagen, Valencia, CA). Protein concentration was assessed with a bicinchoninic acid (BCA) assay according to the manufacturer's instructions (Thermo Fisher Scientific), and purity was assessed by SDS‐PAGE and Western Blot (Supporting Information Figure S1).

### 2.3 Nanoparticle synthesis and characterization

The 270‐nm OVA PNP cores were made as previously described.^34^ Briefly, 0.4 ml pure ethanol was added at a constant rate to 0.1 ml of 6.2 mg/ml OVA in PBS under constant stirring at 600 rpm. The amine‐reactive crosslinker 3,3′‐dithiobis[sulfosuccinimidylpropionate] (DTSSP) (ThermoFisher Scientific) was used to stabilize the resulting nanoparticles. The nanoparticles were cross‐linked in 0.82 mM DTSSP while stirring at room temperature for 1 hr, followed by centrifugation to collect the particles and resuspension in PBS by sonication.

OVA PNP cores were coated with FliC by resuspension in 0.9 mg/ml FliC in PBS, and stirred at 600 rpm overnight at 4°C. Coated particles were collected by centrifugation, and resuspended in 5.26 μM DTSSP to stabilize the adsorbed coat. After stirring at 600 rpm for 1 hr at 4°C, the cross‐linking reaction was quenched with 50 mM Tris base, and the particles were resuspended by sonication in PBS.

OVA PNP cores were coated with IgM by affinity immobilization. One hundred microgram of OVA PNP cores were mixed with 17.5 μg of anti‐OVA mouse IgM (Chondrex, Redmond, WA) in 0.1 ml PBS, and stirred at 4°C for 30 min. Binding was quenched by the addition of 24 μg soluble OVA, and the particles were collected by centrifugation and resuspended by sonication in PBS.

Nanoparticle size distribution and zeta potential were assessed by dynamic light scattering and electrophoretic light scattering, respectively, with a Malvern Zetasizer Nano ZS (Malvern Instruments, Westborough, MA). Nanoparticle concentration was assessed with a BCA assay according to the manufacturer's instructions (Thermo Scientific). Nanoparticles were resuspended in water, air‐dried, and sputter‐coated with palladium prior to visualization with a Zeiss Ultra60 FE (Carl Zeiss Microscopy, Cambridge, UK) scanning electron microscope at 5.0 kV.

### 2.4 IgM coating characterization

IgM coating was confirmed by a standard enzyme‐linked immunosorbent assay (ELISA) procedure. Briefly, 0.2 μg/ml OVA‐IgM PNPs in PBS were incubated on ELISA plates overnight at room temperature. IgM concentration was evaluated using a standard curve of anti‐OVA IgM. Samples were blocked with 1% bovine serum albumin (BSA) in PBS, and probed with an horseradish peroxidase (HRP)‐conjugated anti‐mouse IgM antibody.

Complement activation was assessed by the MicroVue CH50 enzyme immunoassay kit (Quidel, San Diego, CA). Human serum was obtained from two, healthy, consenting donors with the approval of Georgia Institute of Technology IRB #H16083. Approximately 20 ml of blood was collected from each donor, and allowed to clot for 30 min at 4°C. Blood was then centrifuged at 2,000×g for 10 min, and the serum decanted off into sterile centrifuge tubes. Serum was stored at 4°C for up to 2 weeks and at −80°C for extended storage. To activate complement, 15 μg of nanoparticles were added to 14 μl serum, and incubated for 1 hr at 37°C. Terminal complement complex (TCC) formation was assessed according to the kit manufacturer's instructions.

### 2.5 FliC coating characterization

FliC activity was characterized by a TLR‐5‐dependent luciferase activation assay in vitro. Hela cells (ATCC, Manassas, VA) were grown in Dulbecco's Modified Eagle's Medium (DMEM) supplemented with 10% fetal bovine serum (FBS), and cultured in humidified 5% CO_2_ at 37°C. Cells were incubated overnight at a density of 2 × 10^6^ cells/well in a 6‐well plate, and transfected the following day with 10 μg pUNO1‐hTLR5, 2 μg pGL4.32‐[Luc2/Nf‐κB/Hygro] (Invivogen, San Diego, CA) and 15 μl Lipofectamine 2000 (Invitrogen, Grand Island, NY) in DMEM with 1% FBS. Transfected cells were plated the following day at a density of 5 × 10^4^ cells/well in a 96‐well plate in DMEM with 1% FBS. Nanoparticles were suspended in fresh DMEM + 1% FBS at a concentration of 1 μg/mL and used to stimulate transfected cells for 8 hr. Bright‐Glo Luciferase Assay reagent (Promega, Madison, WI) was diluted 1:1 with serum‐free DMEM and used to assess luciferase activity according to the manufacturer's instructions.

### 2.6 Immunization

All animal work was compliant with the NIH Guide for the Care and Use of Laboratory Animals and all protocols and procedures employed were reviewed and approved by the Emory University Institutional Animal Care and Use Committee. Seven‐week old female Balb/c mice (Jackson Laboratory, Bar Harbor, ME) were given 50 μl intramuscular (i.m.) injections into the right hind‐leg of 0.2 mg/ml nanoparticle formulations as described in Table 1. Injections were repeated 21 days after priming for a boost administration.

**Table 1:** Immunization and sample collection schedule for the various groups tested

	Prime day 0	Bleed day 14	Boost day 21	Bleed day 36	Sacrifice day 39
G1: OVA‐OVA NPs					
G2: OVA‐FliC NPs					
G3: OVA‐IgM NPs					
G4: OVA NPs + sFliC					
G5: OVA‐FliC and OVA‐IgM					
G6: PBS					

*Note*. Each group had five mice. Flic = flagellin; IgM = immunoglobulin M; OVA = ovalbumin; NP = Nanoparticles; PBS = phosphate‐buffered saline; sFlic = soluble FliC.

### 2.7 Sample collection

Blood was collected from immunized mice by submandibular venipuncture 2 weeks after prime and boost immunizations. Blood was allowed to clot at 4°C for at least 30 min, and was centrifuged at 5,000 rpm for 5 min to collect serum. Serum samples were stored at −20°C for further analysis.

Following euthanasia on Day 39, splenocytes were prepared from mouse spleens. Briefly, spleens extracted from mice were homogenized manually with the plunger of a 1 ml syringe and cells collected by centrifugation at 300×g for 5 min. Cells collected were resuspended in red blood cell lysis buffer (150 mM NH_4_Cl, 10 mM NH_4_HCO_3_, 1 mM Na_2_EDTA, pH 7.4) for 5 min at room temperature, quenched with RPMI 1640 media (ATCC, Manassas, VA) and centrifuged for 5 min at 2,300×g. Splenocytes collected were resuspended in RPMI 1640 at 4°C and counted by flow cytometry (BD Accuri c6, BD Biosciences, San Jose, CA).

### 2.8 Serum antibody assessment

OVA‐specific IgG antibody titers were assessed by ELISA, as previously described.^10^ Briefly, serial twofold dilutions of serum were analyzed using a standard ELISA procedure, with 1 μg/ml OVA in PBS as the capture antigen, 1% BSA in PBS as the blocking solution, and 1 μg/ml HRP‐anti‐mouse IgG in 1% BSA solution as the detection antibody. Chromogenic quantification was assessed by the oxidation of tetramethylbenzidine by hydrogen peroxide (R&D Systems, Minneapolis, MN) according to the manufacturer's instructions. Two times the absorbance of naïve group's serum samples was considered the cutoff for measuring the endpoint titer.

OVA‐specific IgG1 and IgG2a concentrations were also assessed by ELISA as described above, using HRP‐conjugated anti‐mouse IgG1 and IgG2a, and monoclonal mouse IgG1‐ or IgG2a‐anti‐OVA to create a standard curve (Chondrex, Redmond, WA).

### 2.9 Cytokine ELISpot

Splenocytes were seeded at a density of 2.5 × 10^6^ cells/ml on interferon γ (IFN‐γ) and interleukin 4 (IL‐4) 96‐well ELISpot membranes (R&D Systems, Minneapolis, MN). Splenocytes were stimulated with or without 50 μg/ml endotoxin‐free OVA, and incubated at 37°C in humidified air with 5% CO_2_ for 36 hr. ELISpot membranes were developed according to the manufacturer's instructions. Wells were imaged using a dissection microscope (Olympus SZX16, Olympus Corporation, Tokyo, Japan), and spots were counted using ImageQuant TL's colony counting software (GE Healthcare, Pittsburgh, PA).

### 2.10 Flow cytometry

Splenocytes were seeded at a density of 2.5 × 10^6^ cells/ml on 96‐well plates, and stimulated with or without 50 μg/ml endotoxin‐free OVA, and incubated at 37°C in humidified air with 5% CO_2_ for 60 hr. Cells were incubated with 1% BSA in PBS overnight at 4°C, and blocked with TruStain FcX anti‐CD16/CD32 (Biolegend, San Diego, CA) at a concentration of 1 μg/10^6^ cells for 1 hr on ice. Alexa‐Fluor 488‐conjugated anti‐CD44 and Alexa‐Fluor 647‐conjugated anti‐CD62L (Biolegend, San Diego, CA) were added to each well at a final concentration of 1 μg/10^6^ cells and 0.25 μg/10^6^ cells, respectively, and incubated on ice for 1 hr. Cells were collected by centrifugation, resuspended in PBS, and analyzed by flow cytometry.

### 2.11 Affinity maturation

Affinity maturation of anti‐OVA serum antibodies was measured using biolayer interferometry with the ForteBio Octet RED96 system (Pall Corporation, Port Washington, NY). Streptavidin Dip‐and‐Read Biosensors were used to immobilize 50 μg/ml biotinylated‐OVA (Axxora Life Sciences, San Diego, CA). OVA‐loaded biosensors were incubated with serum samples diluted 1:50, 1:100, and 1:200 in PBS for 5 min, followed by a 5‐min incubation in PBS to measure *k*_on_ and *k*_off_, respectively. The resulting binding curves were analyzed using the Octet Data Analysis software package Version 9.0.0.4 to determine *K*_D_ values.

### 2.12 Statistical analysis

Serum antibody titers were analyzed using the Mann–Whitney *U* test. Antibody concentrations and T cell counts were analyzed using one‐way analysis of variance (ANOVA) followed by Sidak's multiple comparisons test. Comparisons between two groups were performed using Student's *t‐*test. All statistical analyses were conducted using GraphPad Prism 6 (GraphPad, La Jolla, CA). The *p* values of *p* < .05 were considered statistically significant (*p < .05, **p < .01). To test our hypotheses, statistical comparisons were assessed between G1 and G3, between G2 and G4, and for T cell counts, between G6 and all other groups. Comparisons between these groups that were significant are noted in the figures, while comparisons that were not significant are not shown.

## 3 Results

### 3.1 Coated PNP synthesis and characterization

**Figure 1:**
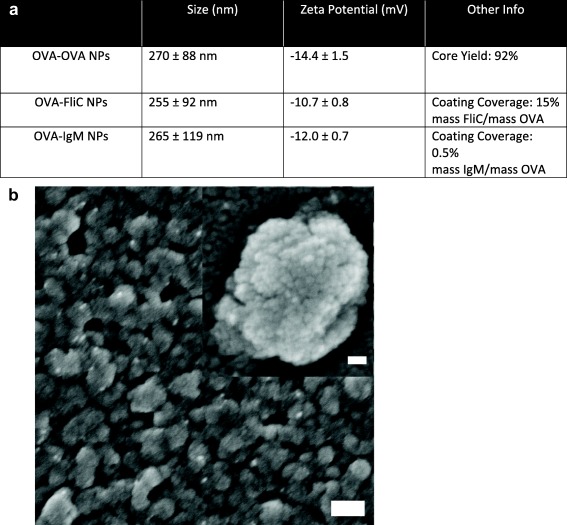
Nanoparticle characterization. (a) Physical characterization data of the different nanoparticles synthesized. (b) Representative scanning electron micrograph of OVA‐coated‐OVA nanoparticles. Outer scale bar, 200 nm. Inset scale bar, 30 nm

Monodisperse, 270 nm OVA nanoparticles were made as previously described.^34^ Coating the nanoparticles did not significantly alter nanoparticle size (Figure 1a). IgM‐coating the nanoparticles without a soluble OVA quenching step resulted in large, 1,000 nm particles, suggesting IgM cross‐linking of multiple nanoparticles (Supporting Information Figure S2). Coating of FliC and IgM on OVA nanoparticles was assessed by FliC supernatant depletion and by anti‐IgM ELISA, respectively. Coverage was reported as an approximate mass adjuvant per mass OVA.

### 3.2 Coat activity

**Figure 2:**
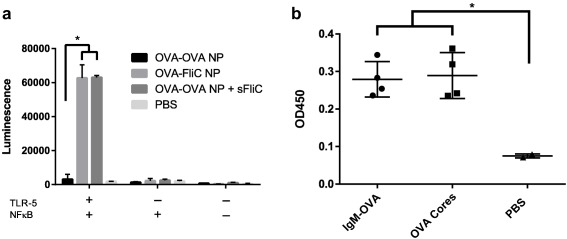
Coat activity was confirmed by in vitro assays specific for each adjuvant. (a) OVA‐FliC nanoparticles and OVA‐OVA nanoparticles with soluble FliC (sFliC) admixed demonstrated similar levels of TLR‐5‐dependent NFκB activation in Hela cells as compared to OVA‐OVA nanoparticles. Each bar is an average of two technical replicates (*n* = 2). (b) Complement activation as determined by anti‐TCC ELISA after mixing nanoparticles with human serum. IgM‐coated OVA nanoparticles and uncoated OVA nanoparticles demonstrated similar levels of complement activation. Each average is composed of two technical replicates in each of two serum samples (*n* = 4)

Coat activity was confirmed by testing FliC and IgM functionality. Since FliC is a TLR‐5 agonist, FliC‐coated nanoparticles were used to activate a TLR‐5‐dependent luciferase assay. FliC‐coated OVA nanoparticles activated TLR‐5 signaling, and did not significantly differ in activity compared to soluble FliC admixed with OVA nanoparticles (Figure 2a). IgM's ability to activate complement was assessed by incubating IgM‐coated nanoparticles with human serum and using ELISA to detect activated complement.^36^ Uncoated OVA nanoparticles were found to activate complement, and the IgM coating on these particles did not significantly enhance complement activation (Figure 2b).

### 3.3 Antibody production

**Figure 3:**
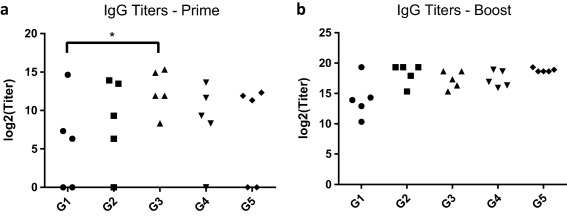
Anti‐OVA IgG titers were assessed 2 weeks after priming (a) and boosting (b) immunizations. Each data point represents the serum dilution factor past which antibody levels were indistinguishable from those in serum of PBS‐immunized mice (G6). Each data point is the average titer of two technical replicates, and titers were assessed for each of the five mice per group. (*p < .05)

Anti‐OVA serum IgG titers were assessed 2 weeks after priming and boosting (Table 1). Following the priming immunization, OVA‐IgM nanoparticles (G3) induced non‐zero responses in all mice, and induced significantly greater responses than OVA‐coated OVA nanoparticles (G1) (Figure 3a). Following a boost immunization of the same formulations, the IgG titers were not significantly different among the groups (Figure 3b). No significant differences in titer were observed between OVA‐FliC nanoparticles (G2) and OVA nanoparticles admixed with soluble FliC (G4).

**Figure 4:**
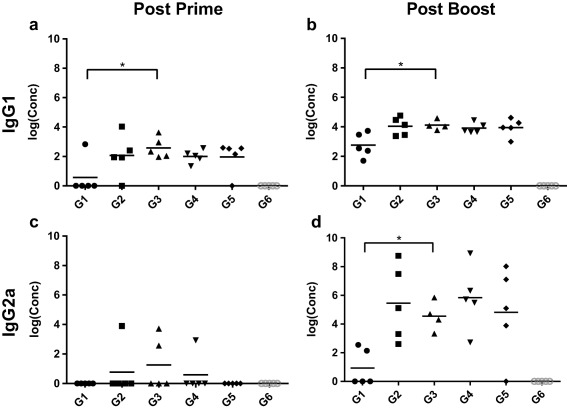
Anti‐OVA serum antibody concentrations of IgG1 and IgG2a, as assessed by ELISA. Each point represents the average concentration as determined by two technical replicates. (*p < .05)

Anti‐OVA IgG subtype concentrations were also assessed after priming and boosting. OVA‐IgM nanoparticles induced significantly higher levels of IgG1 than OVA‐OVA nanoparticles did after both priming and boosting (Figure 4a,b). Appreciable IgG2a responses only appeared after the boost immunization in all adjuvanted nanoparticle groups (Figure 4c,d). No significant differences were observed between OVA‐FliC nanoparticles and OVA nanoparticles admixed with soluble FliC.

### 3.4 T cell cytokines

**Figure 5:**
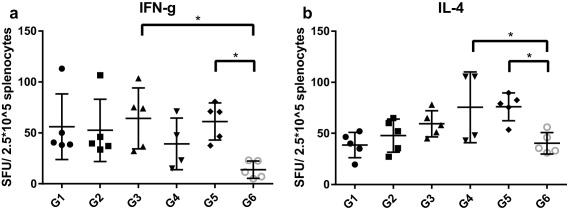
IFN‐γ (a) and IL‐4 (b) ‐secreting T cell counts in 2.5 × 10^5^ splenocytes post‐stimulation with 50 μg/ml OVA. Each data point is an average of two technical replicate counts

ELISpot was used to examine the ability of OVA‐stimulated splenocytes from immunized mice to produce IFN‐γ and IL‐4. Both OVA‐IgM (G3) and OVA‐FliC + OVA‐IgM (G5) immunized mice produced significant amounts of IFN‐γ‐secreting splenocytes (Figure 5a). OVA + sFliC (G4) and OVA‐FliC + OVA‐IgM (G5) immunized mice produced significant amounts of IL‐4 (Figure 5b).

### 3.5 Memory T cells

**Figure 6:**
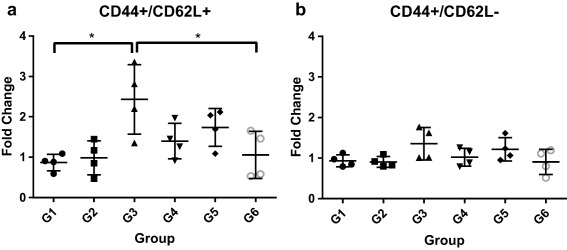
Fold change of CD44^+^/CD62L^+^ (a) and CD44^+^/CD62L^−^ (b) splenocytes after stimulation with 50 μg/ml OVA. Each data point is the ratio of the average number of positive cells in a stimulated versus an unstimulated sample of splenocytes. Each average was derived from two technical replicate samples of 10,000 cells each. Example gating is shown in Supporting Information Figure S3

OVA‐stimulated and unstimulated splenocytes were stained for CD44 and CD62L and assessed by flow cytometry to profile memory T cell activation. CD44^+^/CD62L^+^ double‐positive T cells are indicative of central memory T cells, while CD44^+^/CD62L^−^ single‐positive cells are indicative of effector memory phenotypes.^37^ Normalizing the number of stimulated, positive cells by the number of unstimulated, positive cells allowed us to report a fold change in the amount of positive cells. We found that OVA‐IgM nanoparticles (G3) induced a significant upregulation of central memory T cells (Figure 6a), and no particle types induced appreciable upregulation of effector memory T cells (Figure 6b).

**Figure 7:**
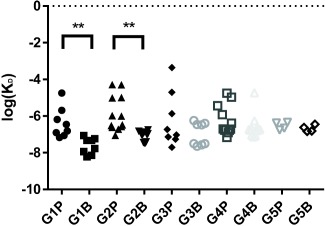
Affinity maturation as assessed by biolayer interferometry. Each point consists of a *K*_D_ value derived from a single association‐dissociation run on the Octet RED96. Each column contains *K*_D_ values obtained from sera from a particular group post‐prime (G#P) or post‐boost (G#B). Replication was assessed over three dilutions of four different serum samples (*n* = 12). Group 5 was assessed at only two dilutions of two serum samples (*n* = 4)

### 3.6 Affinity maturation

Anti‐OVA antibody affinity was measured with the Octet RED system. Average log(*K*_D_) values for post‐prime and post‐boost sera were compared to test for affinity maturation. Significant affinity maturation was found in mice immunized with OVA‐OVA nanoparticles (G1) and OVA‐FliC (G2) nanoparticles (*p* < .01) but not in mice immunized with OVA‐IgM nanoparticles (G3), OVA‐OVA + sFliC (G4), or OVA‐IgM+ OVA‐FliC (G5).

## 4 Discussion

Our previous work with OVA nanoparticles highlighted the importance of protein nanoparticle coating in altering dendritic cell inflammatory responses.^34^ In addition to coating our nanoparticles with antigen, the current study explores the in vivo immune responses to pathogen‐ and host‐derived adjuvant coatings on to nanoparticles.

### 4.1 Flagellin‐mediated adjuvancy

When OVA‐FliC nanoparticles (G2) and OVA nanoparticles admixed with soluble flagellin (G4) were used to immunize mice, both groups developed similar levels of anti‐OVA IgG titers (Figure 3) and serum anti‐OVA IgG1/IgG2a concentrations (Figure 4). The production of high IgG2a levels after the boost immunization is consistent with other literature showing FliC on nanoparticles generates a T_H_1‐biased response.^17^ However, the most drastic difference between the two forms of FliC adjuvant presentation was that affinity maturation of anti‐OVA serum antibodies was triggered by G2, but not by G4.

The phenomena of affinity maturation and class switching have classically been reported in the literature to occur in parallel upon immunization or infection.^7^ To the best of our knowledge, only recently have the two phenomena been studied independently of one another.^38^ Our observation that different modes of FliC presentation lead to differences in affinity maturation while not affecting class switching to IgG2a supports growing evidence that adjuvant presentation method can influence the resulting immune response.^39^ Further work should examine whether affinity maturation is mediated by surface presentation of other TLR‐based adjuvants on nanoparticles as well, since nanoparticles can facilitate ligand access to certain intracellular TLRs. A broader study of soluble versus nanoparticle‐bound TLR ligands can also address whether stimulation of antigen‐internalizing antigen‐presenting cells (APCs) or downstream immune effector cells is more immunogenic on an adjuvant‐by‐adjuvant basis.

### 4.2 IgM as a host‐derived adjuvant

Potential safety issues have been raised for TLR ligand‐based adjuvants that may dissociate or diffuse away from the antigen.^6^ Unlike FliC, host‐derived IgM that may dissociate from the nanoparticles is probably not going to be seen as immunogenic as soluble FliC, and thus an OVA nanoparticle + soluble IgM group was not included in the study design.

Antibodies have been proposed as host‐derived adjuvants before.^33^, ^40^ Most of these studies have been with soluble immune complexes consisting of soluble antigen bound to a cognate antibody.^41^, ^42^ This strategy targets the antigen to Fc receptor‐bearing antigen presenting cells, yet does not exploit a second feature of antibody‐mediated adjuvancy—the activation of complement.

Complement activation can be triggered by the proximity of two IgG Fc domains, or one IgM Fc domain exposed upon antigen binding.^32^ Activation of complement is necessary for vaccination not only as an innate host defense mechanism,^7^ but also for bridging innate and adaptive immune responses.^43^ Triggering complement activation may further enhance the potency of immunoglobulin‐adjuvanted vaccines, and the nanoparticle antigen delivery platform is well‐suited to mediate this effect.

The IgM‐coated OVA particles (G3) did not trigger significant complement activation as compared to uncoated OVA nanoparticles (Figure 2a). While strategies for enhancing IgM density on the particle surface may increase the likelihood of stronger complement activation, OVA‐IgM nanoparticles still significantly enhanced antibody and T cell responses even in the absence of complement activation.

Anti‐OVA IgG endpoint titers significantly increased after one immunization with OVA‐IgM nanoparticles (G3), as compared to unadjuvanted OVA nanoparticles (G1). Following the boost immunization, OVA‐IgM nanoparticles induced elevated levels of IgG2a, whereas unadjuvanted OVA nanoparticles (G1) did not. Unexpectedly, unadjuvanted OVA nanoparticles induced affinity maturation of antibodies, whereas OVA‐IgM nanoparticles only triggered IgG2a antibody class switching and not affinity maturation. The inverse relationship between these phenomena has, to the best of our knowledge, never been reported before.

The strong IgG2a responses elicited by IgM were supported by the high levels of IFN‐γ‐producing T cells, both indicators of a strong T_H_1 response. The T_H_1 and T_H_2 responses are mutually inhibitory,^44^ and during many infections, one response can be protective while the other can be fatal. The T_H_1 response is induced in response to viral and bacterial infections,^7^ and therefore priming a T_H_1‐biased T cell response with antiviral and antibacterial vaccines is critical for successful immunization.

As successful vaccination requires immunological memory, the generation of memory T cell responses is crucial. CD44 and CD62L can be used to identify central memory T cells (T_CM_, CD44^+^/CD62L^+^) and effector memory T cells (T_EM_, CD44^+^/CD62L^−^).^20^ OVA‐IgM nanoparticles stimulated the strongest T_CM_ differentiation (Figure 6a) of all the nanoparticle formulations, supporting the case for IgM as an adjuvant for promoting cell‐mediated immunity. However, none of the nanoparticle formulations induced strong T_EM_ responses (Figure 6b), indicating that the nanoparticles and adjuvants used were unable to completely polarize the T cell response to a T_H_1 or T_H_2 response.^45^

### 4.3 Summary

In this work, we tested the efficacy of a host‐derived adjuvant, IgM, as well as the use of a pathogen‐derived adjuvant both on nanoparticles and admixed with them. Our results are summarized in Table 2. Our FliC‐coated nanoparticles elicited comparable antibody titers to other FliC‐adjuvanted nanovaccines.^17^, ^46^ In the group combining both OVA‐FliC and OVA‐IgM particles (G5), we saw high IFN‐γ production characteristic of G3, low central memory T cell production characteristic of G2, and high IL‐4 production, which was uncharacteristic of either component nanoparticle alone. Although the benefits of combining these two types of adjuvanted nanoparticles are not immediately obvious, there is a synergistic effect as evidenced by the IL‐4 response. Other work has shown that delivery of two types of adjuvants in separate particles elicits greater effects compared to adjuvant co‐delivery in the same particle.^9^

**Table 2:** Summary of immune responses to different adjuvants on OVA nanoparticles

Nanoparticle type	IgG1	IgG2a	IFN‐γ	IL‐4	CD44^+^/CD62L^+^ T cells	Affinity maturation
OVA‐OVA	Low	Low	No	No	No	Yes
OVA‐FliC	High	High	No	No	No	Yes
OVA‐IgM	High	High	Yes	No	Yes	No
OVA + soluble FliC	High	High	No	Yes	No	No
OVA‐FliC + OVA‐IgM	High	High	Yes	Yes	No	No

*Note*. T cell responses of “Yes” and “No” have been made with respect to the PBS (G6) control. Flic = flagellin; IgM = immunoglobulin M; IgG = immunoglobulin G; OVA = ovalbumin; IFN‐γ = interferon γ; IL‐4 = interleukin 4.

Perhaps our most surprising finding was that antibody affinity maturation and IgG2a class switching did not correlate with one another. While the two processes are normally associated with each other in the development of an antibody response,^7^ we found that unadjuvanted OVA nanoparticles and FliC‐coated OVA nanoparticles triggered affinity maturation, while IgM‐ and soluble FliC‐adjuvanted nanoparticles did not. Our results stand in contrast to those by Corley et al., who showed that IgM‐bound soluble antigen (IgM‐ICs) accelerates affinity maturation responses to T‐dependent antigens.^47^ Future work should examine the differences in immune responses to soluble and nanoparticulate immune complexes, and whether such a difference can be exploited to tune the affinity of the humoral immune response. Affinity maturation is necessary for generating high affinity, neutralizing antibodies, which can be protective against highly conserved pathogens.^48^ For pathogens that mutate or change yearly, such as influenza, however, the generation of high‐affinity neutralizing antibodies results in a loss of antibody diversity, and can contribute to the phenomenon known as original antigenic sin, in which antibodies are only made to epitopes found on the first strain of virus the immune system encountered.^7^ If vaccine adjuvants can delay the affinity maturation process while promoting diversification of antibody effector functions via class switching, it is possible that the memory B cell repertoire generated from the immunization will be more effective at combatting rapidly mutating pathogens.

## 5 Conclusion

As vaccination moves away from the isolate‐inactive‐inject paradigm^49^ and toward more engineered vaccine formulations for directing the immune response, the interplay between particulate and molecular adjuvants needs to be understood. We examined the role of adjuvant location on model OVA PNPs with flagellin, and found that FliC location directs the affinity maturation response. To sidestep potential issues with pathogen‐derived adjuvant toxicity, we also explored using immunoglobulins as a host‐derived, immunostimulatory adjuvant coating on nanoparticles. We found that although IgM coating on OVA nanoparticles does not significantly enhance complement activation in vitro, it does enhance antibody and memory T cell responses in vivo, while not promoting affinity maturation. Further studies need to be done to investigate the effector functions of other classes of immunoglobulin adsorbed to nanoparticles, and if the delayed affinity maturation responses we see with our vaccine nanoparticles can translate to protective immune responses in in vivo challenge models of highly mutable pathogens.

## Supporting information

Additional Supporting Information may be found online in the supporting information tab for this article.

Supporting Information

## References

[1] Leleux J , Roy K. Micro and nanoparticle‐based delivery systems for vaccine immunotherapy: An immunological and materials perspective. Adv Healthc Mater. 2013;2(1):72–94. 23225517 10.1002/adhm.201200268

[2] Brewer JM , Pollock KGJ , Tetley L , Russell DG. Vesicle size influences the trafficking, processing, and presentation of antigens in lipid vesicles. J Immunol. 2004;173(10):6143–6150. 15528351 10.4049/jimmunol.173.10.6143

[3] Lundqvist M , Stigler J , Elia G , Lynch I , Cedervall T , Dawson KA. Nanoparticle size and surface properties determine the protein corona with possible implications for biological impacts. Proc Natl Acad Sci USA. 2008;105(38):14265–14270. 18809927 10.1073/pnas.0805135105 PMC2567179

[4] Kumar S , Anselmo AC , Banerjee A , Zakrewsky M , Mitragotri S. Shape and size‐dependent immune response to antigen‐carrying nanoparticles. J Control Release. 2015;220(pt A):141–148. 26437263 10.1016/j.jconrel.2015.09.069

[5] Beningo KA , Wang YL. Fc‐receptor‐mediated phagocytosis is regulated by mechanical properties of the target. J Cell Sci. 2002;115(4):849–856. 11865040 10.1242/jcs.115.4.849

[6] Irvine DJ , Swartz MA , Szeto GL. Engineering synthetic vaccines using cues from natural immunity. Nat Mater. 2013;12(11):978–990. 24150416 10.1038/nmat3775 PMC3928825

[7] Murphy K , Travers P , Walport M , Janeway C. Janeway's Immunobiology. New York: Garland Science; 2012.

[8] Fearon DT , Locksley RM. Elements of immunity ‐ The instructive role of innate immunity in the acquired immune response. Science. 1996;272(5258):50–54. 8600536 10.1126/science.272.5258.50

[9] Kasturi SP , Skountzou I , Albrecht RA , et al. Programming the magnitude and persistence of antibody responses with innate immunity. Nature. 2011;470(7335):543–547. 21350488 10.1038/nature09737 PMC3057367

[10] Wang L , Hess A , Chang TZ , et al. Nanoclusters self‐assembled from conformation‐stabilized influenza M2e as broadly cross‐protective influenza vaccines. Nanomedicine. 2014;10(2):473–482. 23988715 10.1016/j.nano.2013.08.005 PMC3948190

[11] Mizel SB , Bates JT. Flagellin as an adjuvant: Cellular mechanisms and potential. J Immunol. 2010;185(10):5677–5682. 21048152 10.4049/jimmunol.1002156 PMC3756556

[12] Kwissa M , Nakaya HI , Oluoch H , Pulendran B. Distinct TLR adjuvants differentially stimulate systemic and local innate immune responses in nonhuman primates. Blood. 2012;119(9):2044–2055. 22246032 10.1182/blood-2011-10-388579 PMC3311246

[13] Lee S , Nguyen MT. Recent advances of vaccine adjuvants for infectious diseases. Immune Network. 2015;15(2):51–57. 25922593 10.4110/in.2015.15.2.51 PMC4411509

[14] Oh JZ , Ravindran R , Chassaing B , et al. TLR5‐mediated sensing of gut microbiota is necessary for antibody responses to seasonal influenza vaccination. Immunity. 2014;41(3):478–492. 25220212 10.1016/j.immuni.2014.08.009 PMC4169736

[15] Kim JR , Holbrook BC , Hayward SL , et al. Inclusion of flagellin during vaccination against influenza enhances recall responses in nonhuman primate neonates. J Virol. 2015;89(14):7291–7303. 25948746 10.1128/JVI.00549-15 PMC4473543

[16] Turley CB , Rupp RE , Johnson C , et al. Safety and immunogenicity of a recombinant M2e‐flagellin influenza vaccine (STF2.4xM2e) in healthy adults. Vaccine. 2011;29(32):5145–5152. 21624416 10.1016/j.vaccine.2011.05.041

[17] Wang BZ , Quan FS , Kang SM , Bozja J , Skountzou I , Compans RW. Incorporation of membrane‐anchored flagellin into influenza virus‐like particles enhances the breadth of immune responses. J Virol. 2008;82(23):11813–11823. 18786995 10.1128/JVI.01076-08 PMC2583664

[18] Salman HH , Irache JM , Gamazo C. Immunoadjuvant capacity of flagellin and mannosamine‐coated poly(anhydride) nanoparticles in oral vaccination. Vaccine. 2009;27(35):4784–4790. 19539576 10.1016/j.vaccine.2009.05.091

[19] ClinicalTrials.gov. U.S. National Institutes of Health : 2016; Database searched for “flagellin” and “fusion”.

[20] Zhang W , Wang L , Liu Y , et al. Immune responses to vaccines involving a combined antigen–nanoparticle mixture and nanoparticle‐encapsulated antigen formulation. Biomaterials. 2014;35(23):6086–6097. 24780166 10.1016/j.biomaterials.2014.04.022

[21] Weimer ET , Ervin SE , Wozniak DJ , Mizel SB. Immunization of young African green monkeys with OprF epitope 8–OprI–type A‐ and B‐flagellin fusion proteins promotes the production of protective antibodies against nonmucoid Pseudomonas aeruginosa. Vaccine. 2009;27(48):6762–6769. 19744586 10.1016/j.vaccine.2009.08.080

[22] Roic B , Cajavec S , Ergotic N , et al. Immune complex‐based vaccine for pig protection against parvovirus. J Vet Med B. 2006;53(1):17–23. 10.1111/j.1439-0450.2006.00907.x 16460351

[23] Rafiq K , Bergtold A , Clynes R. Immune complex‐mediated antigen presentation induces tumor immunity. J Clin Invest. 2002;110(1):71–79. 12093890 10.1172/JCI15640 PMC151032

[24] Fossati G , Bucknall RC , Edwards SW. Insoluble and soluble immune complexes activate neutrophils by distinct activation mechanisms: Changes in functional responses induced by priming with cytokines. Ann Rheum Dis. 2002;61(1):13–19. 11779751 10.1136/ard.61.1.13 PMC1753889

[25] Kim MY , Reljic R , Kilbourne J , et al. Novel vaccination approach for dengue infection based on recombinant immune complex universal platform. Vaccine. 2015;33(15):1830–1838. 25728317 10.1016/j.vaccine.2015.02.036

[26] Getahun A , Dahlstrom J , Wernersson S , Heyman B. IgG2a‐mediated enhancement of antibody and T cell responses and its relation to inhibitory and activating Fc gamma receptors. J Immunol. 2004;172(9):5269–5276. 15100265 10.4049/jimmunol.172.9.5269

[27] Hjelm F , Carlsson F , Getahun A , Heyman B. Antibody‐mediated regulation of the immune response. Scand J Immunol. 2006;64(3):177–184. 16918684 10.1111/j.1365-3083.2006.01818.x

[28] Clarke CA , Kulke W , Krevans JR , et al. Further experimental studies on prevention of Rh haemolytic disease. Br Med J. 1963;1(5336):979–984. 14021558 10.1136/bmj.1.5336.979 PMC2122888

[29] Gunawan C , Lim M , Marquis CP , Amal R. Nanoparticle‐protein corona complexes govern the biological fates and functions of nanoparticles. J Mater Chem B. 2014;2(15):2060–2083. 10.1039/c3tb21526a 32261489

[30] Cruz LJ , Rueda F , Cordobilla B , et al. Targeting nanosystems to human DCs via Fc receptor as an effective strategy to deliver antigen for immunotherapy. Mol Pharm. 2011;8(1):104–116. 21121669 10.1021/mp100178k

[31] Sorman A , Zhang L , Ding ZJ , Heyman B. How antibodies use complement to regulate antibody responses. Mol Immunol. 2014;61(2):79–88. 25001046 10.1016/j.molimm.2014.06.010

[32] Rosse WF. Quantitative immunology of immune hemolytic anemia: I. The fixation of C1 by autoimmune antibody and heterologous anti‐IgG antibody. J Clin Invest. 1971;50(4):727–733. 4995527 10.1172/JCI106543 PMC291986

[33] Ilag LL. Immunoglobulin M as a vaccine adjuvant. Med Hypotheses. 2011;77(4):473–478. 21723670 10.1016/j.mehy.2011.06.013

[34] Chang TZ , Stadmiller SS , Staskevicius E , Champion JA. Effects of ovalbumin protein nanoparticle vaccine size and coating on dendritic cell processing. Biomater Sci. In Press. DOI: 10.1039/C6BM00500D 10.1039/c6bm00500d PMC5285395 27918020

[35] Chaung HC , Cheng LT , Hung LH , et al. Salmonella flagellin enhances mucosal immunity of avian influenza vaccine in chickens. Vet Microbiol. 2012;157(1‐2):69–77. 22226542 10.1016/j.vetmic.2011.12.014

[36] Pacheco PM , Le B , White D , Sulchek T. Tunable complement activation by particles with variable size and Fc density. Nano Life 2013;3(2):1341001. 24009645 10.1142/S1793984413410018 PMC3759286

[37] Baron V , Bouneaud C , Cumano A , et al. The repertoires of circulating human CD8+ central and effector memory T cell subsets are largely distinct. Immunity. 2003;18(2):193–204. 12594947 10.1016/s1074-7613(03)00020-7

[38] Gitlin AD , von Boehmer L , Gazumyan A , Shulman Z , Oliveira TY , Nussenzweig MC. Independent roles of switching and hypermutation in the development and persistence of B lymphocyte memory. Immunity. 2016;44(4):769–781. 26944202 10.1016/j.immuni.2016.01.011 PMC4838502

[39] Manmohan S. Vaccine Adjuvants and Delivery Systems. Hoboken, NJ: Wiley‐Interscience; 2007.

[40] Getahun A , Heyman B. How antibodies act as natural adjuvants. Immunol Lett. 2006;104(1‐2):38–45. 16364455 10.1016/j.imlet.2005.11.005

[41] Hioe CE , Visciano ML , Kumar R , et al. The use of immune complex vaccines to enhance antibody responses against neutralizing epitopes on HIV‐1 envelope gp120. Vaccine. 2009;28(2):352–360. 19879224 10.1016/j.vaccine.2009.10.040 PMC2789659

[42] Janczy JR , Ciraci C , Haasken S , et al. Immune complexes inhibit IL‐1 secretion and inflammasome activation. J Immunol. 2014;193(10):5190–5198. 25320279 10.4049/jimmunol.1400628 PMC4225162

[43] Ghannam A , Pernollet M , Fauquert JL , et al. Human C3 deficiency associated with impairments in dendritic cell differentiation, memory B cells, and regulatory T cells. J Immunol. 2008;181(7):5158–5166. 18802120 10.4049/jimmunol.181.7.5158

[44] Mosmann TR , Sad S. The expanding universe of T‐cell subsets: Th1, Th2 and more. Immunol Today. 1996;17(3):138–146. 8820272 10.1016/0167-5699(96)80606-2

[45] Sallusto F , Geginat J , Lanzavecchia A. Central memory and effector memory T cell subsets: function, generation, and maintenance. Annu Rev Immunol. 2004;22:745–763. 15032595 10.1146/annurev.immunol.22.012703.104702

[46] Dakterzada F , Mohabati Mobarez A , Habibi Roudkenar M , Mohsenifar A. Induction of humoral immune response against *Pseudomonas aeruginosa* flagellin(1‐161) using gold nanoparticles as an adjuvant. Vaccine. 2016;34(12):1472–1479. 26868080 10.1016/j.vaccine.2016.01.041

[47] Corley RB , Morehouse EM , Ferguson AR. IgM accelerates affinity maturation. Scand J Immunol. 2005;62:55–61. 10.1111/j.1365-3083.2005.01610.x 15953185

[48] Germain RN. Vaccines and the future of human immunology. Immunity. 2010;33(4):441–450. 21029956 10.1016/j.immuni.2010.09.014

[49] Poland GA , Ovsyannikova IG , Kennedy RB , Haralambieva IH , Jacobson RM. Vaccinomics and a new paradigm for the development of preventive vaccines against viral infections. OMICS. 2011;15(9):625–636. 21732819 10.1089/omi.2011.0032 PMC3166201

