# Supplementary material for: Host‐ and pathogen‐derived adjuvant coatings on protein nanoparticle vaccines
Source: Bioeng Transl Med. 2017 Feb 3;2(1):120–30. doi: 10.1002/btm2.10052 (PMC5412930; doi:10.1002/btm2.10052)
Supplement: Supplementary file 1 — Supporting Information [file BTM2-2-120-s001.docx]

Supplemental Information

| 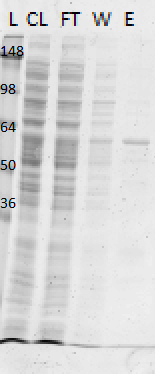S1A | 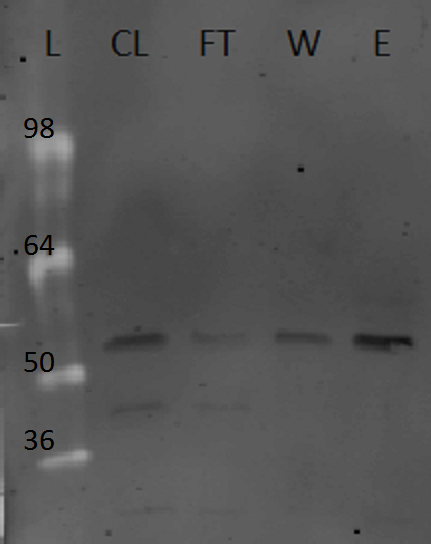S1B |
| --- | --- |
| Figure S1. SDS-PAGE and Western blots run after Ni-affinity purification of recombinant FliC. The elution fraction of the FliC purification shows a single band between 64 and 50 kDa (S1A). A western blot was stained using a 488-Conjugated Penta-His antibody (Qiagen, Valencia, CA), and confirmed that FliC was present between the 64 kDa and 50 kDa ladder bands (S1B). | |

| 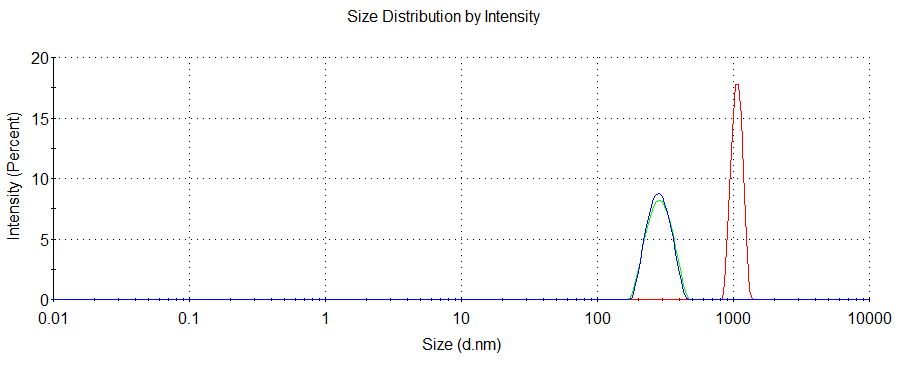S2 |
| --- |
| Figure S2. Preliminary coating of OVA nanoparticles (green) with anti-OVA IgM resulted in the formation of microparticles (red), suggesting crosslinking of OVA nanoparticles by the pentameric IgM. Quenching the coating process by addition of soluble OVA following IgM resulted in no change in nanoparticle size (blue). |

|  |  |
| --- | --- |
| Splenocytes from PBS control mice(G6) | Splenocytes from IgM-NP-immunized mice |

Figure S3. Example gating used on splenocytes from immunized mice. FL1-A = CD44 signal, FL4-A = CD62L signal. Numbers of cells considered double-positive in calculations for figure 6 were counted from the upper right quadrant of all samples.
